# CAPIH: A Web interface for comparative analyses and visualization of host-HIV protein-protein interactions

**DOI:** 10.1186/1471-2180-9-164

**Published:** 2009-08-12

**Authors:** Fan-Kai Lin, Chia-Lin Pan, Jinn-Moon Yang, Trees-Juen Chuang, Feng-Chi Chen

**Affiliations:** 1 Division of Biostatistics and Bioinformatics, Institute of Population Health Sciences, National Health Research Institutes, Miaoli, 350 Taiwan, Republic of China; 2 Genomics Research Center, Academia Sinica, Taipei, 115 Taiwan, Republic of China; 3 Department of Life Science, National Chiao-Tung University, Hsinchu, 300 Taiwan, Republic of China; 4 Department of Dentistry, Chinese Medical University, Taichung, 404 Taiwan, Republic of China

## Abstract

**Background:**

The Human Immunodeficiency Virus type one (HIV-1) is the major causing pathogen of the Acquired Immune Deficiency Syndrome (AIDS). A large number of HIV-1-related studies are based on three non-human model animals: chimpanzee, rhesus macaque, and mouse. However, the differences in host-HIV-1 interactions between human and these model organisms have remained unexplored.

**Description:**

Here we present CAPIH (Comparative Analysis of Protein Interactions for HIV-1), the first web-based interface to provide comparative information between human and the three model organisms in the context of host-HIV-1 protein interactions. CAPIH identifies genetic changes that occur in HIV-1-interacting host proteins. In a total of 1,370 orthologous protein sets, CAPIH identifies ~86,000 amino acid substitutions, ~21,000 insertions/deletions, and ~33,000 potential post-translational modifications that occur only in one of the four compared species. CAPIH also provides an interactive interface to display the host-HIV-1 protein interaction networks, the presence/absence of orthologous proteins in the model organisms in the networks, the genetic changes that occur in the protein nodes, and the functional domains and potential protein interaction hot sites that may be affected by the genetic changes. The CAPIH interface is freely accessible at http://bioinfo-dbb.nhri.org.tw/capih.

**Conclusion:**

CAPIH exemplifies that large divergences exist in disease-associated proteins between human and the model animals. Since all of the newly developed medications must be tested in model animals before entering clinical trials, it is advisable that comparative analyses be performed to ensure proper translations of animal-based studies. In the case of AIDS, the host-HIV-1 protein interactions apparently have differed to a great extent among the compared species. An integrated protein network comparison among the four species will probably shed new lights on AIDS studies.

## Background

Cross-species virus infections usually raise serious threats to worldwide public health. Examples include the Acquired Immunodeficiency Syndrome (AIDS) and Avian Influenza, where viruses cross species boundaries to infect humans from simians [1], and birds [2], respectively. The interactions between virus and host proteins are essential to the completion of virus life cycle, and impact directly on the pathology of infectious diseases [3-6]. Therefore, studies of host-virus interactions are critical to understanding of the virology and development of therapeutics for viral diseases. Since host switching, host specificity, and disease severity all depend on host-virus interactions, comparative studies of host-virus interactions may help unravel the host/viral factors key to these central themes in infectious disease studies.

As one of the most deadly diseases to humans, AIDS is barely life-threatening to chimpanzees [7], human's closest relative in the nature. Comparative studies have provided clues to the differential susceptibility to AIDS between the two species [8-10]. However, since human and chimpanzee protein sequences are almost identical in most of the cases [11], the amino acid substitutions that may lead to *Homo-Pan *divergences in protein-protein interactions (PPIs) have remained elusive. Nevertheless, it is feasible to pinpoint species-specific post-translational modifications (PTMs), which are known to affect host-virus protein interactions [12,13] and can be altered by minor genetic changes such as single nucleotide substitutions or small insertions/deletions (indels). PTMs are pivotal to a wide range of biological processes, including signal transduction, protein targeting, receptor specificity, and PPIs [14]. So far, comparative tools for exploring the potential influences of species-specific PTMs on host-virus interactions have not been found.

Here we develop a web-based interactive database – CAPIH (Comparative Analysis of Protein Interactions for HIV-1) – for comparative studies of genetic differences between the human proteins involved in host-HIV protein interactions and their orthologues retrieved from three mammalian species: chimpanzee (*Pan troglodyte*), rhesus macaque (*Macaca mulatta*), and mouse (*Mus musculus*). The three latter species are all important animal models for HIV studies [15-17]. Understanding the differences in host-virus interplay between human and the model species is the basis for correct interpretation of animal-based HIV studies. Furthermore, by comparing protein interactions between species, one can potentially identify key differences that underlie chimpanzee resistance to AIDS. To facilitate inter-species comparisons of host-HIV PPIs, four main functions are provided in CAPIH. Firstly, the interface shows the presence or absence of orthologous proteins, thus enabling users to pinpoint missing protein components in the host-HIV interaction network. Secondly, the multiple sequence alignments of orthologous proteins enable users to identify species-specific amino acid substitutions, nucleotide substitutions, and indels. This information is helpful for inferring functional changes of orthologous proteins. Thirdly, predictions of 7 types of species-only PTMs (phosphorylation, methylation, sumoylation, acetylation, sulfation, N-glycosylation, and O-glycosylation) for each HIV-interacting host protein are presented for analyses of potential PTM influences on protein interactions and signal/regulatory pathway. We also collect experimentally verified PTMs in human proteins. Fourthly, CAPIH shows potential PPI hot sites on the multiple sequence alignments. Through the visualized interface, researchers can easily spot multiple host factors that directly or indirectly interact with the same HIV protein, and consider how changes in one member protein may affect the protein interaction network.

### Construction and content

#### CAPIH organization and implementation

**Figure 1:**
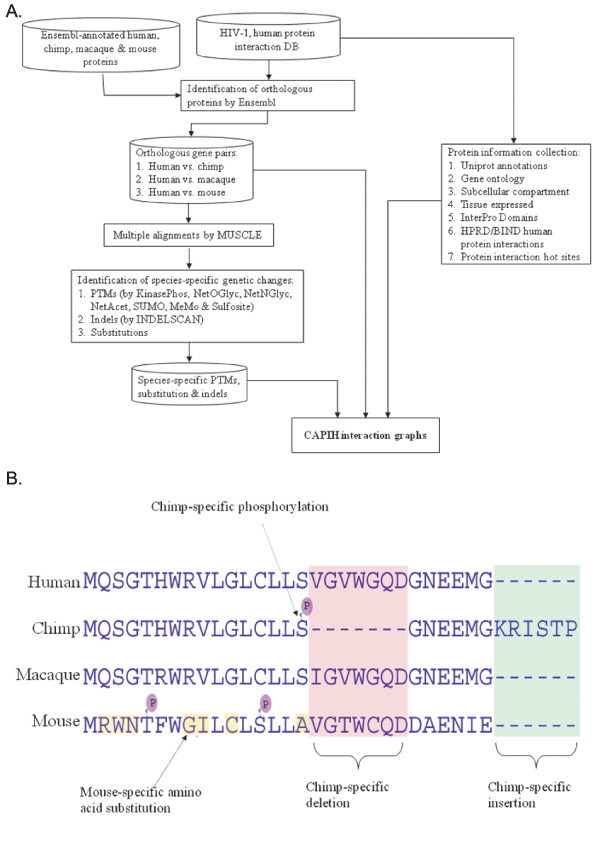
**(A) The data compiling process of CAPIH**. (B) The definitions of species-specific genetic changes. A species-specific genetic change must be an event that occurs in only one out of at least three sequences. Note that the sequences in this figure are modified from real sequences.

The data compiling process is illustrated in Figure 1A. We retrieved a total of 1,447 HIV-1 interacting human proteins from the HIV-1, Human Protein Interaction Database [18] (the November 13, 2007 freeze). The human-chimpanzee-macaque-mouse orthologous proteins were downloaded from the Ensembl genome browser (release 47), which were identified by the Ensembl project using the Markov clustering algorithm [19]. Note that not all the retrieved human proteins have orthologues in all of the three compared species. In the cases of one-to-many/many-to-many orthologous relationships, only the protein pairs with the reciprocally highest similarity were selected. All of the protein and nucleotide sequences were downloaded from Ensembl. Also note that, among the 1,447 retrieved human proteins, 77 proteins had no Ensembl-identified orthologues in any of the compared species. The remaining 1,370 orthologous protein groups were subsequently aligned using the MUSCLE multiple sequence alignment package [20]. Based on the protein alignments, the corresponding transcripts were aligned and separated into 3 types of genomic regions: 3'UTRs, 5'UTRs and coding sequences (CDSs). Since CAPIH aims to identify species-specific genetic changes (Figure 1B), only orthologous genes from at least three species were considered. In this interface, species-specific indels were identified by using the INDELSCAN Web server [21,22]. Meanwhile, CAPIH shows 7 types of species-specific PTM sites, which were identified by 7 well-known PTM prediction packages with default parameters (including MEMO [23], SUMOsp [24], NetOGlyc [25], NetNGlyc, SulfoSite [26], and NetAcet [27]; Table 1). Considering the relatively low quality of chimpanzee and macaque genomic sequences, we used the Phred quality score of 25 as a cutoff to filter out potential false positive predictions. The quality scores of chimpanzee and macaque genomic sequences were downloaded from the UCSC genome browser [28]. In the case of indels, the quality scores of the 15 nucleotides on either side of the indel were averaged. Whereas in the case of PTMs, 15 nucleotides on either side (i.e. 5 amino acid residues) plus the three nucleotides of the PTM-affected amino acid were taken into account. The potential protein interaction hot sites were identified using 3D-partner [29].

**Table 1:** The PTM prediction tools used in the study.

PTM types	Tools	Web sites	Ref.
methylation	MEMO	http://www.bioinfo.tsinghua.edu.cn/%7Etigerchen/memo/form.html	[23]
phosphorylation	KinasePhos	http://kinasephos.mbc.nctu.edu.tw/	[24]
sumoylation	SUMOsp	http://bioinformatics.lcd-ustc.org/sumosp/prediction.php	[25]
O-glycosylation	NetOGlyc	http://www.cbs.dtu.dk/services/NetOGlyc	[25]
N-glycosylation	NetNGlyc	http://www.cbs.dtu.dk/services/NetNGlyc	NA
sulfation	SulfoSite	http://sulfosite.mbc.nctu.edu.tw	[26]
acetylation	NetAcet	http://www.cbs.dtu.dk/services/NetAcet	[27]

Since the HIV-human protein interactions encompassed a wide variety of interaction types, we classified these interactions into 7 major groups based on 65 key phrases from the HIV-1, Human Protein Interaction Database: (1) physical interaction; (2) regulatory interaction; (3) post-translational modification; (4) transportation; and (5) positive interaction (6) negative interaction (7) others. The classification of interaction key phrases can be found online at http://bioinfo-dbb.nhri.org.tw/capih/keyphrases.php?search_target=keyphrases. Note that these key phrases are retrieved from different publications. Consequently, a "biological interaction" may be represented by more than one key phrases. For instance, protein A may "bind" and "inhibit" protein B. In addition, to extend the depth of the visualized network, we also incorporated interactions between human proteins downloaded from the BIND [30] and HPRD databases [31].

#### Species-specific genetic changes identified by CAPIH

The numbers of species-specific genetic changes identified by CAPIH are shown in Tables 2 and 3. Collectively, the interface has identified more than 86,000, 21,000, and 33,000 species-specific amino acid substitutions, indels, and PTM events, respectively, in the four species. For lineage-specific PTM events, in general, phosphorylation account for the largest proportion of all PTM events, followed by glycosylation (O- and N-linked types together), methylation, sulfation, sumoylation, and lastly by acetylation (Table 3). We find that rhesus macaque has a much larger number of species-specific PTM events than hominoids, whereas human and chimpanzee have approximately equal numbers. Since the annotations of chimpanzee and rhesus macaque genes have remained incomplete, we are conservative about the estimates of the numbers of species-specific PTMs. For accuracy, we further exclude the PTM events that occur in indels (including both lineage- and non-lineage-specific indels), all the numbers of lineage-specific PTMs are thus decreased dramatically (Table 3). Nevertheless, each of the hominoids still has more than 950 species-specific PTM events, and rhesus macaque has ~4,600. This observation is consistent with the primate phylogeny. Considering that chimpanzee is highly resistant to developing AIDS while the other two are not, it is of great interest to investigate whether these PTM events play important roles in AIDS development after HIV-1 infections.

**Table 2:** The numbers and distributions of species-specific substitutions and indels

Type	Location	Species			
		Human	Chimp	Macaque	Mouse
Nucleotide Substitution	3' UTR	3,948	2,242	7,256	133,503
	5' UTR	1,343	1,237	2,276	23,082
	CDS (amino acids)	5,675 (1,575)	5,329 (1,449)	35,285 (13,704)	261,565 (69,378)
**Subtotal**		**10,966**	**8,808**	**44,817**	**418,150**

Indels	3' UTR	441	293	1,002	10,883
	5' UTR	210	205	443	2,037
	CDS (amino acids)	331 (145)	711 (325)	1,998 (770)	2,805 (1,914)
**Subtotal**		**982**	**1,209**	**3,443**	**15,725**

**Table 3:** The numbers of species-specific PTMs.

PTM Type	Human	Chimp	Macaque	Mouse
Phosphorylation	1,182 (633)	1,271 (602)	4,111 (3,266)	17,748 (14,789)
Methylation	201 (86)	240 (117)	594 (460)	1,768 (1,403)
O-Glycosylation	262 (142)	222 (135)	643 (467)	1,778 (1,409)
N-Glycosylation	60 (43)	60 (39)	193 (167)	716 (614)
Sulfation	68 (51)	72 (50)	212 (186)	573 (509)
Sumoylation	43 (19)	31 (15)	114 (94)	569 (466)

Acetylation	11 (4)	23 (10)	39 (25)	66 (42)

**Total**	**1,827 (978)**	**1,919 (968)**	**5,906 (4,665)**	**23,218 (19,232)**

The numbers of PTMs that occur outside of indels are shown in the parentheses.

#### Description of the CAPIH Web interface

**Figure 2:**
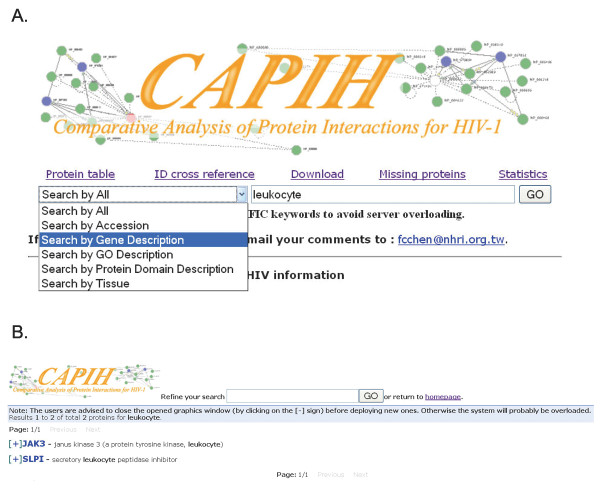
**(A) The query schemes of CAPIH**. (B) All the proteins that match the query key word will be shown with a plus "+" sign in front. Detailed information of each protein can be shown by clicking on the "+" sign.

**Figure 3:**
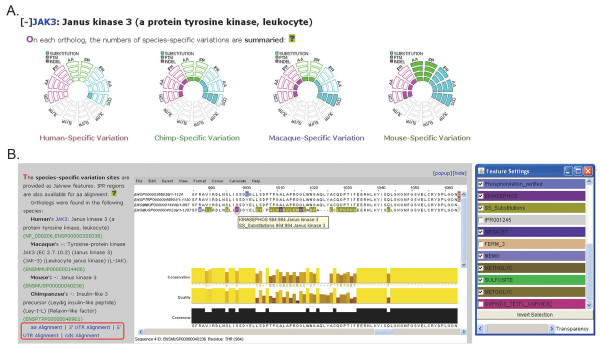
**(A) Statistics of species-specific changes in different regions**. Each filled block represents ~10 species-specific genetic changes. AA: amino acid; IPR: Interpro-predicted protein domain; CDS: coding sequence; 3/5 UTR: 3'/5' untranslated regions. (B) Multiple amino acid sequence alignment wherein species-specific changes (PTMs, and substitutions) and InterPro domains are shown in colored boxes. Indels are not color-shaded. The colors can be shown or hidden by checking the boxes in the "Feature Settings" panel. The user can obtain more information of each color shaded amino acid residue by pointing the cursor to the residue of interest. Shown in the figure is a mouse-specific phosphorylation event predicted by KinasePhos at position 984. The user can also choose to view the nucleotide sequence alignments in 5'/3' UTR or coding sequence by clicking on the hyperlinks in the left panel.

**Figure 4:**
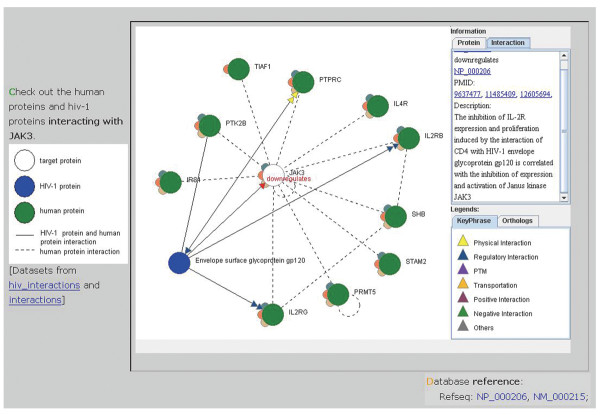
**An example of HIV-human protein interaction graph**. The white, blue, and green circles represent the target, HIV-1, and other human proteins, respectively. Information of any of the protein can be obtained on the right panel by clicking on that protein circle. The triangles each represent a PPI key phrase based on one research article. By clicking on one of the triangles, the users can obtain more detailed information on the right panel, including a short description of the interaction, a PubMed hyperlink to the original publication, and hyperlinks to the annotations of the interacting proteins. The dashed lines indicate HPRD- and BIND-based interactions between human proteins. The circled dashed lines indicate self-interactions. The semi-circles around each protein node indicate the presence of orthologous proteins in the non-human organisms. The entire graph can be zoomed in and out by holding and moving the right mouse click. The graph can also be moved along by holding and moving the left mouse click.

The CAPIH interface provides five query schemes: by gene accession number, gene description, gene ontology, protein domain, and expressing tissue (Figure 2A). Alternatively, the user can also look up the proteins of interest in the protein table, which includes all the proteins analyzed in the interface. All the proteins that match the query key word will be shown with a plus "+" sign in front (Figure 2B). Detailed information of each protein can be shown by clicking on the "+" sign (Figures. 3 and 4). Note that the information page of each protein is composed of three sections ("Genome Comparison Statistics", "Multiple Sequence Alignments", and "Protein Interactions"). By default only the first section will be deployed when the page is shown. The user can deploy the other two sections by clicking the "+" sign before each section. The user can further refine the search by submitting a second key word, or return to the homepage and start a new search. For each protein of interest, CAPIH shows the statistical pie diagram of species-specific variations in the "Genome Comparison Statistics" section (substitutions in light blue, indels in purple, and PTMs in green color; Figure 3A). For substitutions and indels, the diagram gives species-specific variations in amino acid sequences, InterPro-predicted protein domains, CDSs, 3'UTR, and 5' UTR (in the top-down direction). Each filled block represents 10 variations. That is, 10 nucleotide substitutions (for CDS and UTRs), amino acid changes (for amino acid and IPR domains), indels, or PTMs. For example, 12 species-specific changes will be shown as 2 filled blocks in the graph. However, if the number of species-specific changes exceeds 40, only 4 filled blocks will be shown (Figure 3A). Note that nucleotide substitutions in coding regions do not necessarily cause amino acid substitutions, whereas indels do. Also note that one indel event may affect more than one amino acids. Therefore, the total numbers of indels and nucleotide substitutions in CDS do not necessarily equal the number of amino acid changes.

The interface also provides an alignment viewer using JalView [32] (The "Multiple Sequence Alignments" section; Figure 3B). JalView helps to show the alignments of orthologous protein, CDS, and UTR sequences, InterPro domains, potential protein interaction hot sites, and species-specific substitutions, indels, and PTMs. All of these features are color-shaded, and can be shown or hidden by changing the check list in the accompanying "Feature Settings" box (Figure 3B). The user can view detailed information of the predicted protein domains and species-specific genetic changes by pointing the cursor to the color-shaded boxes. Note that the features may overlap with each other. Therefore, some features may not be seen unless the overlapping features are hidden. The users are advised to take advantage of the Feature Settings box to obtain a clear view of the sequence alignment. A detailed description of JalView can be found at the JalView website http://www.jalview.org.

CAPIH also provides a JAVA-based adjustable protein interaction viewer (The "Protein Interactions" section; Figure 4). The interaction view gives the user an idea of how HIV-1 proteins interact with the proteins of interest. To extend the scope of interactions, we also include human protein interactions downloaded from the BIND and HPRD databases [30,31], in addition to HIV-1-human protein interactions. The BIND and HPRD interactions are shown in dashed lines, whereas the HIV-1-human protein interactions in solid lines with colored triangles representing different interaction types. HIV-1 proteins, human proteins, and the protein of interest are shown in blue, green, and white circles, respectively. In this interaction graph, user adjustment is allowed. Any one of the circles can be selected by a mouse click. The selected protein then turns red and can be dragged along with the cursor. Clicking on the blank region will release it. The graph can also be dragged along by clicking and holding the left mouse click, or be zoomed in/out by using the right click in the same way. CAPIH also provides protein IDs and detailed descriptions of interactions when the users click on the corresponding part of the graph. The protein IDs and reference PubMed IDs are hyperlinked to the corresponding databases for more detailed information. An online help file can be found at http://bioinfo-dbb.nhri.org.tw/capih/help.php?search_target=help. The identified species-genetic changes are downloadable at http://bioinfo-dbb.nhri.org.tw/capih/download_table.php?search_target=download.

### Utility

#### Example 1

It has been suggested that changes in T cell surface glycans may be associated with *Homo-Pan *differences in CD4^+ ^T cell-mediated immune responses against HIV infection [10]. It is therefore of interest to investigate the differences in glycosylation between human and the other model organisms. From CAPIH, we have identified 322 and 282 human- and chimpanzee-only glycosylation events, respectively (Table 3). Many of these proteins are T cell surface antigens. For example, CAPIH shows two experimentally verified N-glycosylation sites in the CD3G molecule (NP_000064) at positions 52 and 92. However, at position 52 the glycosylation site (Asn) was substituted by Thr in mouse, whereas the one at position 92 becomes Asp and Glu in rhesus macaque and mouse, respectively. Therefore, human has one and two more N-glycosylation sites, separately, when compared with rhesus macaque and mouse. These glycosylation sites are interesting targets for experimental verification and subsequent functional analyses. If the glycosylation events are proven important for changes in immune responses, researchers can further examine CD3G-related PPIs to explore the underlying molecular mechanisms.

#### Example 2

Another example involves the well-known group of restriction factors, the APOBEC proteins. CAPIH includes 6 members of this group, namely APOBEC3A, 3B, 3C, 3D, 3F, and 3G. CAPIH indicates that none of these proteins has an orthologue in the mouse genome. Since the APOBEC3 proteins are known to be involved in host defense against retroviruses, these proteins have undergone substantial changes because of positive selection [33,34]. This is a good example of remarkably different host factors even between very closely related species such as human and chimpanzee. Indeed, CAPIH identifies a considerable number of genetic changes in the cytidine deaminase domains of the human-chimpanzee APOBEC3 orthologues (Table 4).

**Table 4:** The genetic changes between human and chimpanzee in the cytidine deminase domains of four APOBEC proteins.

	APOBEC3A	APOBEC3B	APOBEC3C	APOBEC3F
A. a substitution	2	11	23	5
PTM	0	7	13	4
Indel	0	0	0	0

Note that APOBEC 3D and 3G are not listed because their human-chimpanzee orthology is ambiguous.

Notably, the mutations in the cytidine deaminase domain are considered responsible for the host-retrovirus PPIs and the host-range specificities of retroviruses [35-37]. It is evident that the APOBEC3 members have experienced very different evolutionary paths in this domain. As shown in Table 4, APOBEC3B and 3C have obviously diverged more than 3A and 3F both in terms of the number of amino acid substitutions and the number of potential PTM changes. It is therefore speculated that APOBEC 3B/3C may have played an important role in the divergence of hominoid immune responses against retroviruses. Nevertheless, the changes in 3A and 3F, though not as drastic, can also have functional effects. Functional studies are required to unravel the biological implications of these changes. Also noteworthy is that no indels are found in the cytidine deaminase domain in all of the four proteins, suggesting strong negative selection on indels in spite of the increased substitution rate in this domain.

#### Example 3

The interaction between human Vpr binding protein (VPRBP) with the HIV-1 Vpr accessory protein is known to be critical for HIV-1 infection ([38]. Inspection of the multiple amino acid sequence alignment of VPRBP reveals that the mouse sequence is shorter than those of the hominoids by nearly 100 amino acid residues at the C-terminus. The C-terminal half of VPRBP has a proline-rich domain and a number of Phe-x-x-Phe repeats, which serves as the Vpr binding domain [39]. Consequently, it is speculated that the loss of the C-terminal amino acids in mouse VPRBP may have certain effects on the Vpr-VPRBP binding affinity. This difference should be experimentally verified, and if proven true, should be taken into account in mouse-based HIV-1 studies.

## Discussion

Here we present the first web-based interactive tool for comparative studies of host-HIV interactions in four different model animals. The interface may provide new insights into HIV studies. Firstly, although mouse is an excellent model for HIV studies, considering the large genetic divergences that occur in protein domains between human and mouse as shown here, many of the host-HIV protein interactions are expected to differ between the two species. Therefore, the differences in genetic backgrounds must be controlled for appropriate interpretations of mouse-based HIV studies.

Secondly, human viral infections transmitted from other species have become a critical issue because humans usually lack the immunological arsenal to fight such viruses [2,40-42]. Comparative studies of host-virus interactions provide a path to understand the possible mechanisms of how viruses break species boundary into humans, and why they cause pathological conditions in humans but rarely do so in other animals. Differences in PPIs may harbor part of the answers. Since PTMs are critical to PPIs, they should be taken into consideration when analyzing the effects of different PPIs on host pathology. Meanwhile, PTM by itself is actually critical to host-virus interactions. Glycosylation, for example, is widely known to be critical to viral recognition and entrance into target cells. Given the wide spectrum of biological functions in which PTMs are involved, variations in host protein PTM patterns should have major impacts on immune response and virus life cycle.

Thirdly, one surprising finding here is that PTMs actually differ to a great extent among the four compared species, considering that they are genetically close to one another. For example, human and chimpanzee differ from each other by an average of two amino acids per protein [11]. In comparison, in the 1,370 proteins compared, human and chimpanzee each has more than 600 species-specific substitution-related phosphorylation sites (Table 3). In other words, on average, each HIV-interacting protein in both human and chimpanzee has an average of 0.4 species-specific phosphorlation sites. This example illustrates the importance of "PTMome". Glycome, the collective sum of all glycans and part of the PTMome (if glycolipids are not considered), is known to be remarkably larger than proteome [43,44]. Therefore, it is easily understandable that PTMome is actually much larger than proteome. The large numbers of species-specific PTMs in HIV-interacting proteins illustrate the great potential of PTM studies in virology and AIDS studies.

## Conclusion

The CAPIH interface is unique because it is the first web-based tool to provide comparative information of genetic changes and PTMs in host-pathogen interactions. Since cross-species viral infections have become a critical issue in public health, comparative studies of host-pathogen interactions deserve wide attention. Specifically, comparative analyses of host-HIV interactions may shed some light on the mechanisms of differences in AIDS progression between human and chimpanzee. A number of possible mechanisms have been proposed [8,45]. However, none of them provides a systematic view in the context of host-HIV protein interactions. Furthermore, PTMs, perhaps one of the most important regulatory mechanisms of host-pathogen protein interactions, have been rarely studied in a comparative way. This interface may provide clues to the potential roles of PTMs in HIV infections, and serve as a starting point for studies on host-HIV protein interaction networks in different hosts.

## Availability and requirements

The CAPIH database is available at http://bioinfo-dbb.nhri.org.tw/hivppi/. The JAVA Runtime Environment is required to view the interactive protein networks.

## Abbreviations

AIDS: acquired immunodeficiency syndrome; CDS: coding sequence; HIV: human immunodeficiency virus; indel: insertions/deletions; PTM: post-translational modification; PPI: protein-protein interaction; UTR: untranslated region.

## Authors' contributions

FCC conceived the project. FKL, CLP, and JMY analyzed the data. FKL and CLP constructed the interface. FCC, FKL and TJC drafted the manuscript. All authors read and approved the manuscript.
